# High-resolution analyses of human sperm dynamic methylome reveal thousands of novel age-related epigenetic alterations

**DOI:** 10.1186/s13148-020-00988-1

**Published:** 2020-12-14

**Authors:** Mingju Cao, Xiaojian Shao, Peter Chan, Warren Cheung, Tony Kwan, Tomi Pastinen, Bernard Robaire

**Affiliations:** 1 Department of Pharmacology and Therapeutics, McGill University, 3655 Promenade Sir William Osler, Montreal, QC H3G 1Y6 Canada; 2 Department of Human Genetics, McGill University, 740 Docteur-Penfield Avenue, Montreal, QC H3A 0G1 Canada; 3 McGill University Genome Quebec Innovation Centre, 740 Docteur-Penfield Avenue, Montreal, QC H3A 0G1 Canada; 4 Digital Technologies Research Centre, National Research Council Canada, 1200 Montreal Road, Ottawa, ON K1A 0R6 Canada; 5 Department of Urology, McGill University Health Centre, 1001 Boulevard Decarie, Montreal, QC H4A 3J1 Canada; 6 Center for Pediatric Genomic Medicine, Children’s Mercy Kansas City, 2401 Gilham Road, Kansas City, MO 64108 USA; 7 Department of Obstetric and Gynecology, McGill University, 1001 Boulevard Decarie, Montreal, QC H4A 3J1 Canada

**Keywords:** Spermatozoa, DNA methylation, Advanced paternal age, MCC-seq, Fertility

## Abstract

**Background:**

Children of aged fathers are at a higher risk of developing mental disorders. Alterations in sperm DNA methylation have been implicated as a potential cause. However, age-dependent modifications of the germ cells’ epigenome remain poorly understood. Our objective was to assess the DNA methylation profile of human spermatozoa during aging.

**Results:**

We used a high throughput, customized methylC-capture sequencing (MCC-seq) approach to characterize the dynamic DNA methylation in spermatozoa from 94 fertile and infertile men, who were categorized as young, 48 men between 18–38 years or old 46 men between 46–71 years. We identified more than 150,000 age-related CpG sites that are significantly differentially methylated among 2.65 million CpG sites covered. We conducted machine learning using our dataset to predict the methylation age of subjects; the age prediction accuracy based on our assay provided a more accurate prediction than that using the 450 K chip approach. In addition, we found that there are more hypermethylated (62%) than hypomethylated (38%) CpG sites in sperm of aged men, corresponding to 798 of total differential methylated regions (DMRs), of which 483 are hypermethylated regions (HyperDMR), and 315 hypomethylated regions (HypoDMR). Moreover, the distribution of age-related hyper- and hypomethylated CpGs in sperm is not random; the CpG sites that were hypermethylated with advanced age were frequently located in the distal region to genes, whereas hypomethylated sites were near to gene transcription start sites (TSS). We identified a high density of age-associated CpG changes in chromosomes 4 and 16, particularly HyperDMRs with localized clusters, the chr4 DMR cluster overlaps *PGC1α* locus, a protein involved in metabolic aging and the chr16 DMR cluster overlaps *RBFOX1* locus, a gene implicated in neurodevelopmental disease. Gene ontology analysis revealed that the most affected genes by age were associated with development, neuron projection, differentiation and recognition, and behaviour, suggesting a potential link to the higher risk of neurodevelopmental disorders in children of aged fathers.

**Conclusion:**

We identified thousands of age-related and sperm-specific epigenetic alterations. These findings provide novel insight in understanding human sperm DNA methylation dynamics during paternal aging, and the subsequently affected genes potentially related to diseases in offspring.

## Background

The current trend in fathering children at an older age has raised concerns due to the reported association between advanced paternal age and increased incidence of several conditions including bipolar disorders, attention deficit hyperactivity disorder (ADHD), and schizophrenia [1]. However, the influence of paternal age on alterations in paternal sperm chromatin is still very limited [2, 3]. Spermatogenesis is a life-long process, and the number of spermatogonial cell divisions prior to spermiogenesis increases from 35 at puberty to more than 600 at 50 years of age [4]. Genetic mutations occur during each replication cycle every 2–3 weeks; the mutational load continuously increases in the sperm of older males [4–6]. In addition to spontaneous genetic mutations during cell division, the epigenetic modifications must be copied to the daughter cells, and copying of the epigenetic information is much more error-prone than DNA replication [7]. The epigenome is an essential and modifiable set of serial marks including DNA methylation, histone posttranslational modifications and small noncoding RNAs. Age-dependent modifications of the germ cells epigenome remain relatively poorly understood.

DNA methylation is a heritable epigenetic modification of cytosine residues within CpG dinucleotides. Among 28 million CpG sites throughout the mammalian genome, approximately 60–80% are methylated, displaying relatively stable DNA methylation patterns in most cell types, except germ cells and pre-implantation embryos [8]. Using a mouse model, Xie et al. identified reduced DNA methylation and differentially methylated promoters in aged sperm, and demonstrated that epigenetic alterations in longevity regulators, reduced life span and more pronounced aging-associated pathologies in offspring of animals sired by old father than by young ones [9]. The relationship between aging and DNA methylation has been studied previously in many human cell types as well as several tissues [10]. Genome-wide methylation studies have revealed that the sperm epigenome differs remarkably from that of somatic cells [8, 11]; a unique state of DNA methylation exists outside of CpG island sequences in mouse sperm DNA [11, 12]. Human sperm DNA methylation is quite distinct from that in all somatic cells and tissues [13]. Earlier studies on sperm DNA methylation were mostly focussed on imprinted loci [14–16]. Only a few studies have examined DNA methylation in human sperm on a genome-wide basis [17–20].

Using the Infinium Human Methylation 450 Bead Chip microarray (450 K), Jenkins et al. have identified 139 regions that are significantly and consistently hypomethylated with age and 8 regions that are significantly hypermethylated with age in human sperm. In addition, a total of 117 genes that are associated with these regions of altered methylation were identified [21]. This research group has also conducted experiments to develop a sperm DNA methylation-based age predicting calculator [22]; their model was able to predict an individual’s chronological age with up to 94% accuracy in comparison with a previously constructed age calculator based on methylation data from somatic tissues [23]. More recently, Denomme et al. applied reduced representation bisulfite sequencing (RRBS) to measure the DNA methylation profiles in sperm of six young (≤ 35 years) and six older men (≥ 50 years), identified 49,792 differentially methylated CpG sites (DMCs) at nominal *p* value or *p* < 0.05 (corresponded to 3,405 sperm differentially methylated regions (DMRs)). They found that methylation alterations are not randomly distributed across the genome and have some links with neurodevelopmental relevant diseases [24]. Despite those recent advances, the information of age-associated sperm DNA methylation is still limited.

Over the past decade, several methods have been developed and applied to characterize DNA methylation at gene-specific loci using either traditional bisulfite-PCR analysis or pyrosequencing [14, 15], or to determine genome-wide distribution using microarray analysis, and RRBS analysis [19]. Although whole genome bisulfite sequencing (WGBS) provides comprehensive coverage of the epigenome [25], it highlights a general inefficiency as 70–80% of the sequencing reads across the in-depth analysis datasets provide little or no relevant information about CpG methylation sites [8]. A commonly used approach to examine human sperm DNA methylation is the 450 K or more recently the Illumina EPIC array chips [21, 26–28]. However, these arrays provide limited coverage of the epigenome, are biased toward genic and CpG-rich regions, and are not specific for the sperm epigenome. MethylC-capture sequencing (MCC-seq) was developed for targeted assessment of DNA methylation in a tissue-specific manner [29], and further customized to target human sperm epigenome with enrichment of sequences of interest in both genic and intergenic regions [20].

The objective of the present study was to identify changes in human sperm DNA methylome as a function of paternal age and fertility status using our previously characterized sperm capture panel [20] in order to enrich bisulfite sequencing coverage to regions of variable and/or dynamic DNA methylation. We also included analyses of the relationship between paternal age and fertility status with total serum and bioavailable testosterone concentrations, serum FSH level, high density lipoprotein (HDL), and metabolic parameters as well as smoking. Using high throughput and customized MCC-seq technique, we identified thousands of age-related epigenomic alterations in human sperm; we also conducted a machine learning model using our dataset to predict the methylation age of subjects. Our results provide new insights on how paternal aging and fertility status interact on an array of male reproductive endpoints and demonstrate novel aspects of how aging modifies the sperm methylation pattern.

## Results

### Subjects descriptions in MCC-seq study cohort

Basic characteristics of subjects in this study are shown in Table 1. Two subpopulations of young (< 40 years, with mean = 29 years) and old (≥ 40 years, with mean = 53 years) subjects were used. Within each group, the fertile control and infertile subjects were matched (except two fewer patients in the infertile group for old group). We compared the general semen parameters, plasma hormone level and key metabolic factors between the two populations. We observed marginally significant changes for sperm motility (49 ± 3% vs. 38 ± 3%, *p* = 0.029) and a significant difference in follicle stimulating hormone (FSH) with lower level in young subjects (4.09 ± 0.29) compared with old subjects (6.25 ± 0.4, *p* = 3.66E−05). We also observed a higher level of testosterone in young subjects than in old ones (11.9 ± 0.6 vs. 9.29 ± 0.52, *p* = 0.001). No differences were found in sperm concentrations, or concentrations of luteinizing hormone (LH), thyroid stimulating hormone (TSH), bioavailable testosterone, total cholesterol, triglycerides, high density lipoprotein (HDL), low density lipoprotein (LDL), cholesterol/HDL risk ratio, body mass index (BMI) and tobacco consumption between young and old groups (Table 1).

**Table 1:** Clinical characterisation of subjects in the MCC-Seq study cohort

Parameter	Young	Old	*p* values
	(mean ± SEM) (*n*)	(mean ± SEM) (*n*)	
Age (years)	29.38 ± 0.91 (48)	53.15 ± 0.98 (46)	**2.04E−31**
Sperm concentrations (× 10^6^/ml)	118.57 ± 15.99 (48)	143.6 ± 18.31 (46)	0.306
Motility (rapid & slow %)	48.96 ± 3.26 (48)	38.77 ± 3.26 (46)	**0.029**
BMI (kg/m^2^)	26.45 ± 0.99 (48)	28.94 ± 0.85 (46)	0.059
FSH (IUs/L)	4.09 ± 0.29 (48)	6.25 ± 0.4 (45)	**3.66E−05**
LH (IUs/L)	3.75 ± 0.17 (48)	4.34 ± 0.32 (45)	0.108
TSH (mIU/L)	2.05 ± 0.34 (47)	1.84 ± 0.21 (42)	0.591
E2RP (qmol/L)	130.97 ± 8.06 (34)	138.24 ± 8.42 (34)	0.535
Testosterone (T, nmol/L)	11.9 ± 0.6 (48)	9.29 ± 0.52 (45)	**0.001**
Bioavailable T (nmol/L)	6.33 ± 0.27 (48)	5.01 ± 0.61 (43)	0.051
Total cholesterol (mmol/L)	5.21 ± 0.79 (48)	5.06 ± 0.44 (45)	0.863
Triglycerides (mmol/L)	1.83 ± 0.37 (48)	2.89 ± 0.74 (45)	0.202
HDL (mmpl)/L	2.58 ± 1.35 (48)	1.87 ± 0.51 (44)	0.624
LDL (mmol/L)	3.05 ± 0.51 (47)	5.68 ± 2.28 (39)	0.265
Cholesterol/HDL risk ratio	3.9 ± 0.2 (47)	4.06 ± 0.17 (43)	0.544
Smoking	*N* (%)	*N* (%)	0.933
Never	29 (61.7%)	28 (61%)	(*χ*^2^ = 0.138)
Past	13 (27.7%)	12 (26%)	
Current	5 (10.6%)	6 (13%)	
NA	1		

Values are shown as the mean ± SEM

*BMI* body mass index, *FSH* follicle stimulating hormone, *LH* luteinizing hormone, *TSH* thyroid stimulating hormone, *HDL* high density lipoprotein, *LDL* low density lipoprotein

### Correlations analysis between the age of subjects and clinical indices in both fertile and infertile men

The relationships between the age of subjects and clinical indices in both fertile and infertile subjects as assessed by Pearson correlation analyses are shown in Additional file 1: Table S1. In fertile men, there were significant positive correlations between the age of the subjects and plasma FSH level, and metabolic parameters such as BMI, plasma triglycerides, and the risk ratio of total cholesterol to HDL. In addition, there were significant negative correlations between the age of subjects and plasma total testosterone and bioavailable testosterone concentrations and high density lipoprotein (HDL). However, in the infertile group many of the correlations were lost; we only found significant negative correlations between the age of subjects and sperm motility, and plasma bioavailable testosterone concentrations (Additional file 1: Table S1).

### Sperm sample purity as assessed by bisulfite pyrosequencing analysis of imprinted loci

Prior to MCC-seq, we screened sperm DNA sample purity by performing bisulfite pyrosequencing of two paternally methylated gene loci H19 and DLK1/GTL2-IG DMR, and two maternally methylated gene loci MEST, KCNQ1OT1. Somatic cell contaminated sperm samples were excluded for further analysis if the aberrant DNA methylation was found. As expected, the DNA methylation profile of those imprinted gene loci obtained from MCC-seq is normal at the QC coordinates at imprinted loci and in turn confirmed our sample purity (Additional file 5: Figure S1).

### MCC-seq detects thousands of novel CpGs associated with sperm aging

**Fig. 1:**
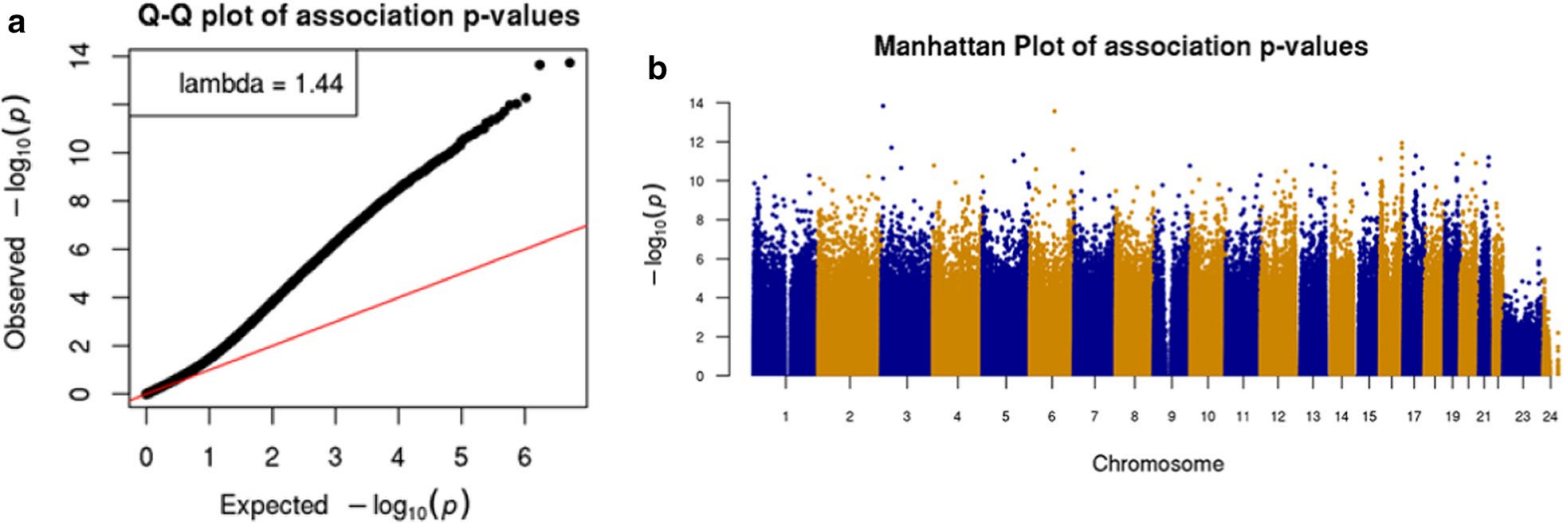
Sperm age EWAS results. **a** CpG—Age association tests QQ-plot shows moderate inflation (GCin = 1.44), and **b** genome-wide abundance of CpGs (> 20 K) passing qv < 0.01 significance threshold

We utilized our previously characterized sperm capture panel [20] to enrich bisulphite sequencing coverage to regions variable in human sperm. We targeted 20–30 × coverage of each (n = 94) donor sample. A minimum 20 × coverage for CpG inclusion in association testing in each and included each targeted CpG with at least 30 samples having data; we note that 80% of targeted CpGs were detected in at least 90% of samples. We initially applied a linear model without covariates and tested association for both fertility and age at 2.65 million passing CpGs. We observed some inflation for statistically significant differences in methylation upon aging genomic control inflation, GCin = 1.7 [30], and less marked statistical inflation for fertility (GCin = 1.39) (Additional file 5: Figure S2). Applying multiple testing correction (q-value, qv < 0.01) no significant CpGs were observed for fertility and > 150,000 significant signals were found for aging. Using a linear model to perform the epigenome-wide associations (EWAS) between age and DNA methylation at 2.65 million passing CpGs with fertility and other age-associated phenotypes (e.g. lipids, BMI, etc., see Methods) as covariates. To this end, after applying multiple testing correction, we observed 21,971 CpGs (*q* value, qv < 0.01) associated with aging with a modest genomic inflation (*G*_in_ = 1.44) (Fig. 1, Table 2).

**Table 2:** Age-associated hypo- and hypermethylated CpGs

	Age qv < 0.01 novel		Age qv < 0.01 in sperm age loci	
All CpGs	Hypermethylated	Hypomethylated	Hypermethylated	Hypomethylated
2599141	13556	8299	6	110

### Specificity of sperm age-associated CpGs

**Fig. 2:**
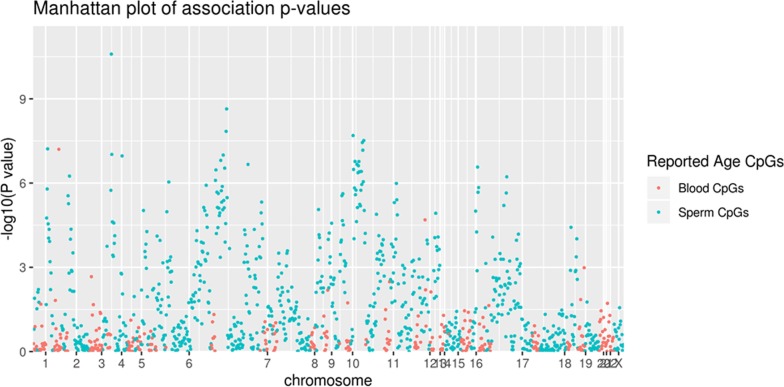
Sperm age associations from MCC-seq are specific. CpG association results for sperm aging intervals reported earlier are shown in green with earlier blood associated CpGs highlighted in red. Only two blood methylation age predicting CpGs reach qv < 0.01, whereas > 100 CpGs in 50 regions are showing significance for sperm methylation age

Since we detected an order-of-magnitude higher number of CpGs associated with sperm aging, we sought to explore their specificity using MCC-seq measured CpGs mapped (*n* = 908) to the 50 genomic regions listed to harbor sperm aging CpGs in a previous study [22]. We observed 116/908 CpGs in 26 of the 50 reported regions reaching qv < 0.01 (417/908 reaching nominal significance *p* < 0.05) for sperm age association. In contrast, for blood aging CpGs [23], we observe only 2 of 307 CpGs for sperm aging at qv < 0.01 (Fig. 2). This tissue restricted 20-fold enrichment of multiple testing corrected associations in our data among previously suggested sperm methylation age signature regions yields strong replication and underscores high specificity of our association; this reiterates the uniqueness for sperm methylation aging as compared to other tissues.

Notwithstanding the strong replication of earlier results, it is intriguing that most of our genome-wide-significant CpGs (> 99.5%) lie in regions not reported previously. We note that only 4.7% of the CpGs associated with aging in our dataset are actually represented on the Illumina 450 K microarray, explaining much of the new discovery stemming from the “dynamic sperm methylome” we specifically targeted by the capture methylome assay [20]. Overall, our results suggest pervasive influence of aging in shaping sperm methylome patterns.

### Chronological age prediction from sperm MCC-seq

**Fig. 3:**
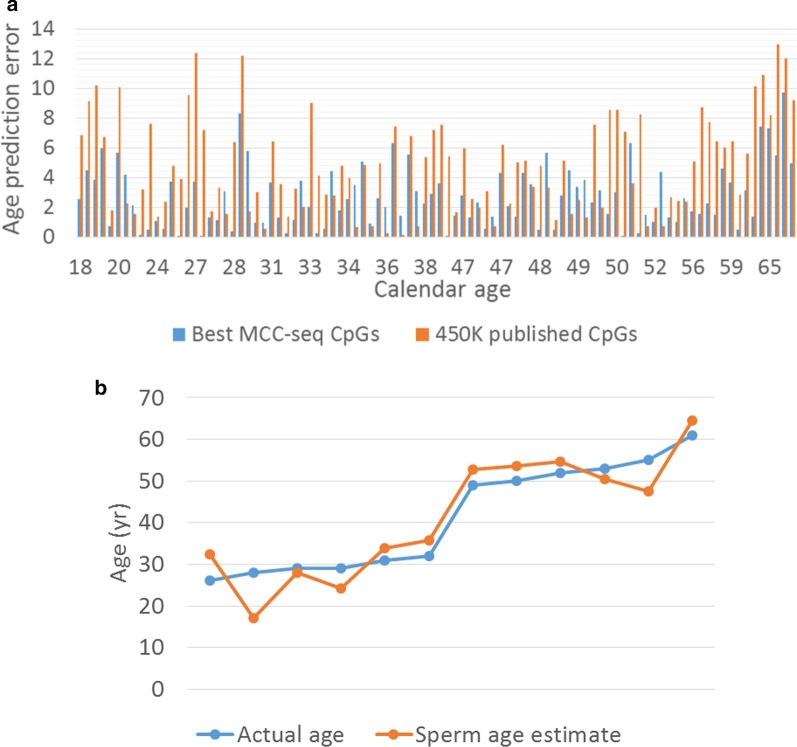
Sperm age prediction analysis. **a** Comparison of age predictor error between model using previously published CpGs detected on Illumina 450 K Human Methylation array (“450 K published CpGs”, orange bars) and the model with top 5000 CpGs (> 99% novel) from our genomewide aging EWAS (“Best MCC-seq CpGs”, blue bars), which provided higher prediction accuracy. **b** Replication of the age predictor in 12 independent samples from our earlier sperm capture methylome study

Next, we explored the prediction of age from sperm methylome patterns. Due to limited training dataset, we trained our models using leave-one-out cross-validation (LOOCV) strategy. That is, each time we leave one sample out and train the model using the remaining samples, and then predict the age for the leave-out sample. The procedure was repeated for each sample. In order to test independence from previously reported sperm age predictor [22], we fetched 450 K CpGs located with Jenkins’ reported sperm aging regions from our sequenced MCC-Seq data (published 450 K sites). By taking advantage of our large panel of MCC-Seq data, we separately trained a model with top 5 K DMCs (> 99% novel age-associated CpGs) detected in the LM age model. In all these cases, the CpGs measured by all samples were eventually used, i.e. CpGs with missing data were removed for training. Finally, as an independent test (replication), we tested our novel sperm age predictor on a recently published sperm cohort [20] where the sperm age predictor was retrained by considering the CpGs measured in all training set (94 samples) and testing set (12 samples). As shown in Fig. 3, the predictor based on novel sites was most accurate with an average error of 2.68 years (years) (Fig. 3a), and, even with limited training set, the replication shows age prediction accuracy with 4.43 year error in the small set of independent samples (*n* = 12) (Fig. 3b). These results underscore the widespread age-related changes in sperm methylome and suggests that age predictors based on sperm methylome alterations can be further refined.

### Characteristics of sperm aging CpGs

**Fig. 4:**
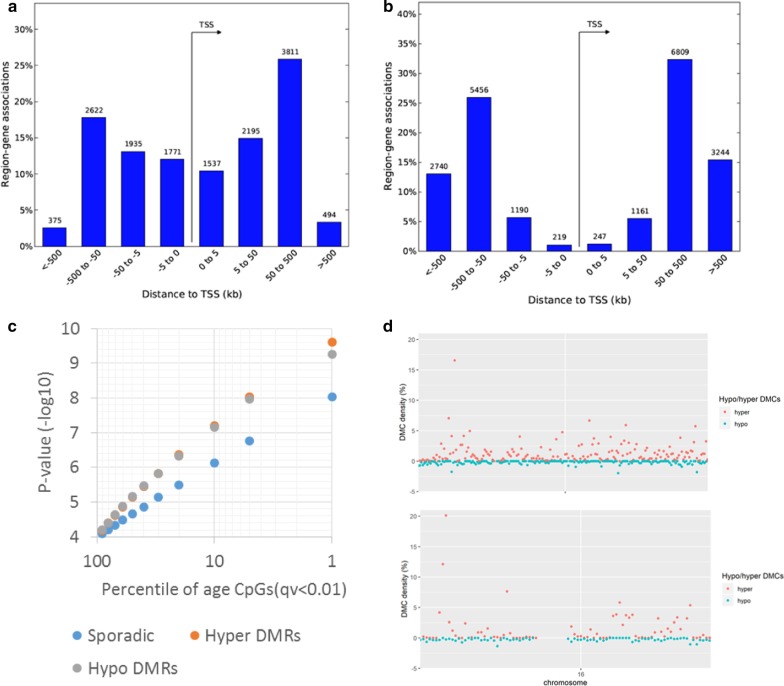
Distribution of age-associated hypo- and hypermethylated CpGs in relation to gene region and in chromosome. **a** Age-associated hypomethylation is proximal to genes, whereas **b** hypermethylation occupies gene distal regions. We used GREAT (PMID 20436461) analyses to annotate each CpG to its closeset transcription start site (TSS). **c** A subset of age-associated CpGs are clustered in differentially methylated regions (DMRs) showing higher significances than sporadic age-associated CpGs. **d** Local density of sperm age-associated hypermethylation (red dots) shows two outlier regions in the genome reaching > 10% density in chr4 and chr16. Density of hypomethylation (green dots) is relatively evenly distributed across the genome. Overall, sex chromosomes are relatively depleted of age-associated CpGs

We first examined the overall genomic distribution of the genome-wide significant age-associated CpGs based on relative hypo and hypermethylation as a function of age. Hypomethylation was observed to be less common (~ 38% of significant sites, *n* = 8409 CpGs) with distribution of CpGs biased strongly towards proximal regulatory elements as compared to more prevalent (~ 62% of significant sites, *n* = 13,562 CpGs) hypermethylation upon aging (Fig. 4a, b). This new observation of predominance of age-associated hypermethylation is solely driven by sperm age-associated regions not detected by Illumina microarrays [21] (Table 2).

Furthermore, we observe that 26% of age-associated CpGs were clustered in DMRs where five or more CpGs in a 500 bp window showed significant association with either hypomethylation (*n* = 315 HypoDMRs) or with hypermethylation (*n* = 483 HyperDMRs) among the total 798 DMRs. The DMRs were characterized by showing stronger associations (Fig. 4c) and particularly hyper DMRs with localized clusters in chromosomes 4 and 16 leading to high density of age CpGs in these loci (Fig. 4d). Of note, chr4 DMR cluster overlaps with the peroxisome proliferator-activated receptor (PPAR)-gamma coactivator (PGC)-1α locus, a protein involved in metabolic aging [31] and chr16 is overlapping RNA binding fox-1 homolog 1 (*RBFOX1)* gene, implicated in neurodevelopmental disease [32].

### The distribution of age-related hypo- or hyper-methylated CpGs in sperm is not random

Using the GREAT [33], we then explored the distribution of age-associated hyper- and hypomethylated CpGs in relation to gene region and to in-chromosome location. We annotated each CpG to its closest transcription start site (TSS), and found that the age-associated hypomethylation is proximal to genes, whereas age-associated hypermethylation occupies gene distal regions (Fig. 4a, b). Furthermore, density of hypomethylation is relatively evenly distributed across the genome. However, overall sex chromosomes are relatively depleted of age-associated CpGs (Additional file 5: Figure S3). Taken together, our results indicate that sperm age-related hypo- or hypermethylated CpGs are not randomly distributed.

### Sperm aging CpGs in relations to chromatin states

The divergence of age-associated hypo- vs. hypermethylated CpGs is also clear from relatively lower average methylation across all samples at age hypomethylated sites 39% ± 20% vs. 60% ± 16% (average ± SD). Consequently, nearly 50% of age-associated hypomethylation localized to open chromatin (DNaseI Hypersensitive Site Master List (125 cell types) from ENCODE/Analysis [34], whereas only 20% age-related hypermethylated sites overlapped open chromatin in multiple human cell types. In line with this observation, the chromatin states enriched strongly for relatively hypermethylated CpGs are strongly biased towards heterochromatin for all ENCODE cell lines; representative distribution of hypo/hypermethylated regions in sperm within Human ES cells (H1/ESC) mapped to chromatin states is shown in Additional file 1: Table S3. Similarly, we observed an enrichment (32% vs. 9%) of CpGs in hypo-DMRs in human sperm H3K27me3 histone modification regions [35] where developmental promoter-like regions were reported in human sperm.

### Analysis of the evolutionarily constrained hyper- and hypomethylated region

**Fig. 5:**
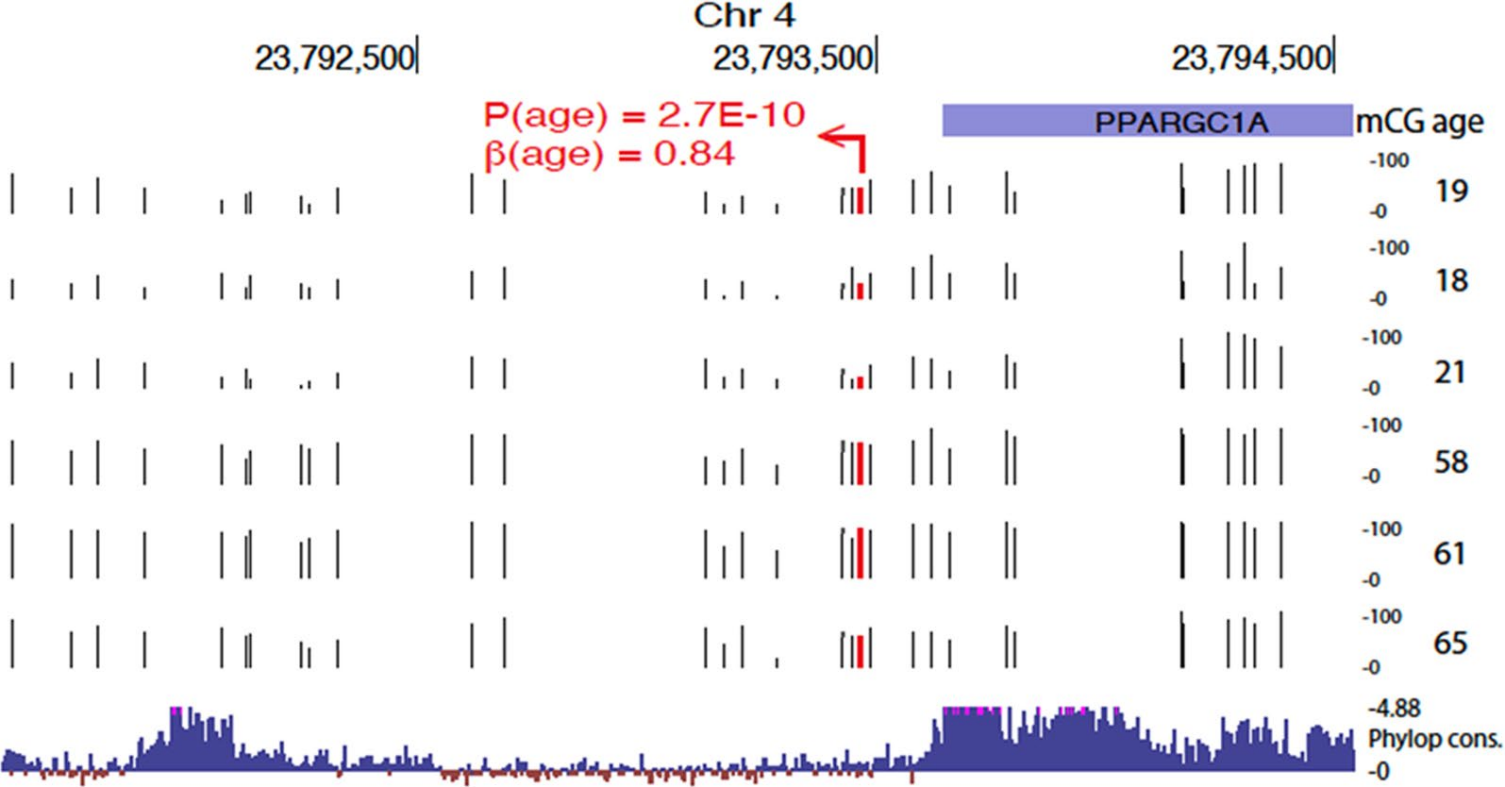
Evolutionarily constrained region 3′ of PGC1α locus in chromosome 4 with cluster of age-induced hypermethylation. Top associated CpG is highlighted in red. Raw methylation values (0–100%) are shown (tracks with black tick marks) for six samples representing extremes of the age distribution in our sample set (ages shown on the right). Bottom track shows 100 vertebrates Basewise Conservation by PhyloP (phyloP100wayAll): high evolutionary constraints are evident by Phylop constraint scores > 4.88 at 3′ end of the transcript and distal intergenic region, spanned by age-related hypermethylation

Genomic element rate profiling (GERP) analysis shows that evolutionarily constraint is similar among hyper- and hypomethylated CpGs (Additional file 5: Figure S4). Particularly, approximately 15% of CpGs lie in areas showing similar constraint as known functional elements (GERP ++ score > 1.7) [36]. However, we also observed regions with high conservation. For example, a HyperDMR spanning over 20 CpGs was observed at the evolutionarily constrained region 3′ of PGC1α locus in chromosome 4 (shown in Fig. 5). Specifically, this high evolutionarily constraint region is evident by Phylop constraint scores > 4.88 at 3′ end of the transcript and distal intergenic region, spanned by age-related hypermethylation where the top CpG within this region shows large effect size of 0.84 (*p* = 2.7e−10).

We further studied the DMRs showing significant evolutionary (GERP ++ score > 1.7) constraint (Additional file 2: Table S4) observing that disease (OMIM) relevant gene loci were slightly less likely to be found among constrained HyperDMRs (61/211, or 29%) as compared to constrained HypoDMRs (39/114 or 34%). However, among HyperDMRs at disease loci we observed primary neuropsychiatric/neurodevelopmental/neurological traits for 31% (19/61), whereas only 10% (4/39) of HypoDMRs at disease loci had primary neurological/neurodevelopmental picture. An example of sperm aging HyperDMR (chr2:166900333–166900544) is overlapping exon 11 of *SCN1A*, most common genetic cause of epilepsy, intriguingly most de novo mutations arise in the paternal chromosome [37], but paternal age has not yet been associated with occurrence of mutations.

### Gene networks implicated by age-associated methylation

**Fig. 6:**
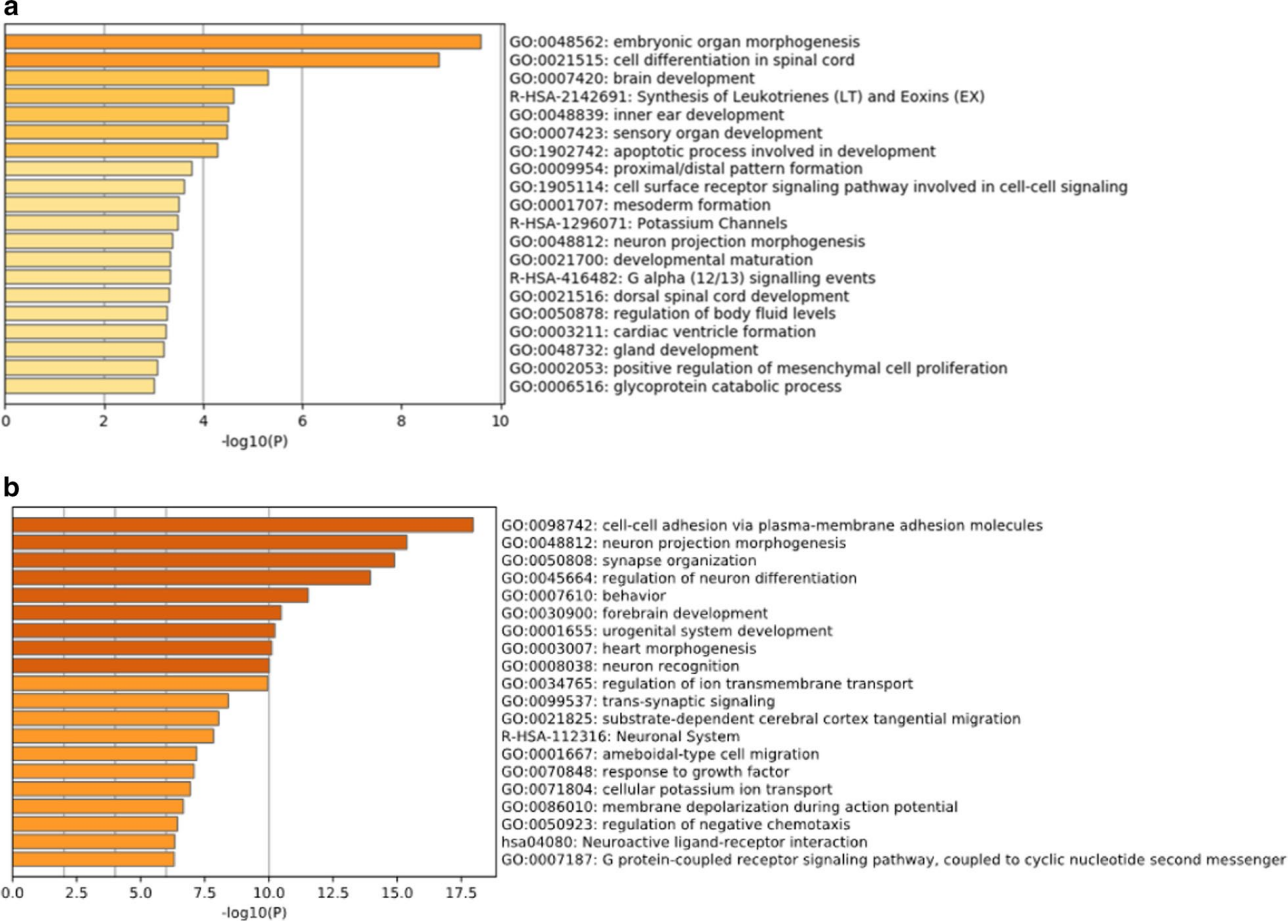
Gene networks implicated by age-associated methylation. **a** Gene ontology (GO) analyses of age genes (*n* = 420) enriched in age dependent hypomethylated regions. **b** GO enrichments in for age dependent genes in hypermethylated regions (*n* = 606)

The CpG enrichments analyses in GREAT were done to look for genes linked to relative hypo- or hypermethylation with higher density of CpG associations in ± 1 Mb for genes than expected by chance (Additional file 3: Table S5). We performed gene ontology (GO) analyses and found an enrichment of 420 genes in age-dependent hypoDMRs, and 606 genes in age dependent hyperDMRs. The top 20 terms were listed for hypoDMRs (Fig. 6a) and hyperDMRs (Fig. 6b); the lists of enriched genes in hyper- and hypomethylated regions are shown in Additional file 4: Table S6. Overall, GO analyses revealed developmental pathways in both hypo- and hypermethylated regions and markedly strong association signals for pathways associated with the central nervous system (CNS) including neuron projection, differentiation and recognition, and behaviour-related terms in hypermethylated regions with age (Fig. 6). It is interesting to note that pathways associated with spermatogenesis or sperm function are not identified in this analysis.

### Weighted gene co-expression network analysis (WGCNA) in sperm MCC-seq

**Fig. 7:**
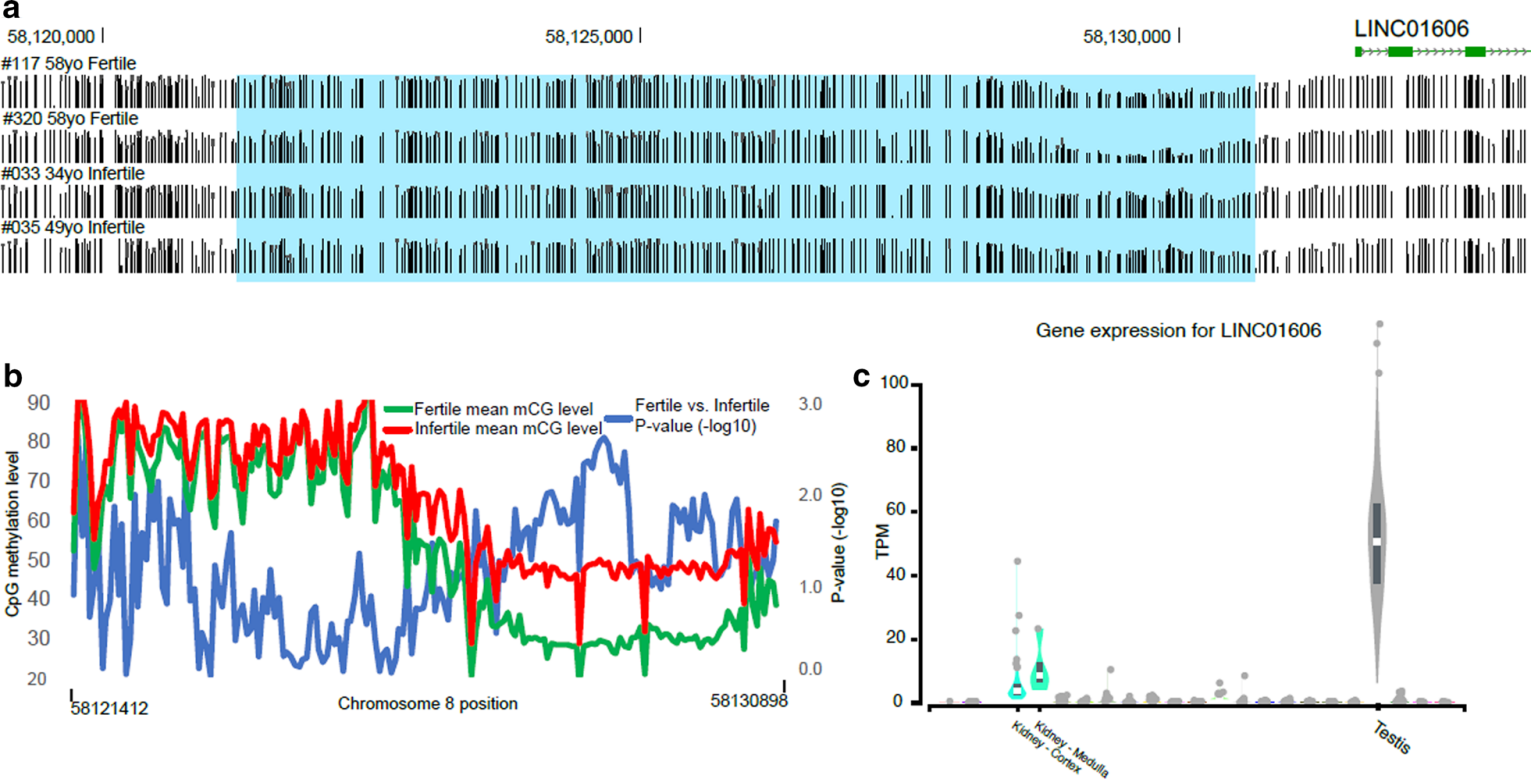
WGCNA revealed a hypermethylated CpG module associated with infertility trait in Chromosome 8 locus. **a** Sperm DNA methylation track pattern was shown in representative fertile and infertile subjects (their ID and age on the left), the hypermethylated CpG module maps proximal to a long noncoding RNA, *LINC01606*. **b** Mean CpG methylation levels of sperm from fertile (green) and infertile (red) men on chr8, p-values were shown in blue (fertile vs. infertile). **c** Gene expression for *LINC01606* in different tissues indicated its testis-selective

WCGNA is an unsupervised method used to identify clusters (modules) of highly correlated features and favours a scale-free network clustering pattern [38]. We have previously utilized this in identifying correlated CpGs in diseases [39]. We reasoned that the approach could identify important sets of CpGs, which may not reach marginal significance in site-wise analyses. We then performed trait correlation against modules identified in WGCNA (Additional file 5: Figure S5). We observed two highly significant module-trait correlations (|AverageCorr|> 0.6) with each linked to age and composed of hundreds of CpGs and involving > 100 gene loci (Additional file 4: Table S6). These large modules (MEblue corr = 0.62, *p* = 2.00E−11 and MEbrown corr = −0.62, *p* = 3.00E−11) show GO enrichments to neuronal as well as developmental processes (Additional file 5: Figure S6) with moderate overlap (> 15%) to marginally significant age-associated CpGs. Several other age trait correlated (|Corr|> 0.3, *p* < 0.005) modules are driven by fewer CpGs and protein coding gene loci most of which were not captured by marginal age associations and may represent important additional loci for aging. In addition, one moderately correlated module (MEtan corr = 0.33, *p* = 0.001) was observed with infertility trait and not detected in pointwise analyses for infertility associations (Additional file 5: Figure S5, Additional file 4: Table S6). Remarkably, the infertility associated CpG module is driven strongly by single chromosome 8 locus (87 of 97 module CpGs), which is hypermethylated in sperm from infertile men (Fig. 7a,b). These CpGs are all in the area of 9 kb region map proximal to testis-selective long-noncoding RNA (*LINC01606*) (Fig. 7c).

## Discussion

To our knowledge, this is the first study that uses a high throughput, customized MCC-seq approach to enrich bisulfite sequencing coverage to regions of variable and/or dynamic DNA methylation in human spermatozoa designed to identify the effects of both increasing paternal age and fertility. In comparison with previously commonly used methods, the merit of higher resolution is that it allows for the identification of thousands of age-related and age-specific epigenetic alterations. In addition, we found that there are more hypermethylated (62%) than hypomethylated (38%) CpG sites during aging. Interestingly, the hypermethylated CpG sites were frequently located in the distal region to genes, whereas hypomethylated sites were often near to gene region. Furthermore, developmental pathways were enriched for both age hypo- and hypermethylated subsets; neuron function and behaviour-related genes were markedly strong and enriched for age hypermethylated regions in spermatozoa. However, these dynamic DNA methylation changes during aging remain to be supported by further experiments using in vivo cell models to detect biological effect on developmental transcription and signaling; transgenerational persistence of these changes could be identified in a longitudinal cohort or in multi-generational families.

We demonstrated that age prediction from our data improved by increasing the number of age-associated regions from earlier reported loci to thousands of novel regions. This suggests that by specific targeting of the full complement of age-associated CpGs and generating deep sequencing data (improving pointwise accuracy of methylation data) across this subset of sperm dynamic methylome could allow for the development of a fine-tuned sperm epigenome age tool.

Our results clearly show that the identified DNA methylation alterations are age-related and specific to germ cells. Sperm DNA methylation patterns were evaluated in 17 fertile donors in a longitudinal study by comparing the sperm methylome of 2 samples collected from each individual 9–19 years apart; in that study, using the Illumina 450 K microarray, 139 age-related hypomethylated regions and 8 age-related hypermethylated regions were identified [21]. In our study (Fig. 2), we found more than 100 age-associated CpGs in previously reported 50 age DMR regions are reaching significance for sperm [22], whereas only two age-related CpGs are significant in blood [23]. This strong replication of previous sperm aging data along with no signal enrichment for blood aging provides clear evidence of sensitivity and specificity of our technology and dataset. Overall, the results suggest that there is a pervasive influence of aging in shaping the sperm methylome patterns.

Other than examining the effects of aging, we also investigated whether the fertility status of these subjects as well as other covariates affected the sperm methylome. Surprisingly, fertility status did not result in significant changes in the DNA methylation pattern. Previous studies indicated that sperm DNA methylation patterns differed significantly with fertility status; > 8500 DMCs were identified and were proposed to be predictive of embryo quality after in vitro fertilization (IVF) [26]. The reasons for the discrepant results between the two studies are not clear. Male infertility is a multi-factorial disorder; infertility of unknown origin accounts for 37–58% of all cases [40]. Although the fertility status of subjects is well defined in this study, and the average sperm concentration was reduced significantly in infertile versus fertile group (Table 1), we did not have severe oligozoospermic men in this study. It has been reported that moderate or severe oligozoospermia is often associated with aberrant DNA methylation of imprinted genes, especially in patient with less than 10 million spermatozoa per ml [41, 42], but the global DNA methylation was normal [14, 43, 44]; this could, in part, explain why we did not find fertility associated DNA methylation alterations in our study cohort.

Although we do not detect fertility associated DNA methylation in pointwise analyses in human sperm, we do observe a moderately correlated CpGs cluster (module) with infertility trait in WGCNA analysis. Specifically, the infertility associated CpG module is hypermethylated on chromosome 8 locus in sperm from infertile men, and is located nearby the upstream of LINC01606, a testis-selective long-noncoding RNA gene. LINC01606 has been shown to be elevated aberrantly and correlated with metastasis and invasion of gastric cancer, and its expression level is associated with Wnt/β-catenin signaling pathway activation through the regulation of miR-423-5P [45]. There is no evidence yet that LINC01606 plays a role in spermatogenesis or male fertility, but whether the hypermethylated CpG module is correlated to this long non-coding RNA gene in infertile men, and which miRNA species are subsequently regulated by LINC01606 in human sperm is worth considering for further study.

The distribution of age-related either hypo- or hyper-methylated CpGs in sperm is not random, as age-associated CpGs in DMRs regions are highly significant (Fig. 4). We identified 798 age-associated DMRs (483 HyperDMRs and 315 HypoDMRs) that are relatively evenly distributed in comparison with only 147 DMRs (8 HyperDMRs and 139 HypoDMRs) with strong bias toward hypomethylation regionally that were identified by Jenkins and colleagues [21]. This suggests that the 450 K array, which has inherent biases with the use of higher density of probes at promoter or gene dense regions, provides a selective perspective on the impact of aging on the methylome.

We identified a high density of age-associated CpGs changes in chromosomes 4 and 16, particularly HyperDMRs with localized clusters. It is interesting to note that the chr4 DMR cluster overlaps *PGC1α* locus, a protein involved in metabolic aging [31] and that chr16 DMR cluster overlaps *RBFOX1* locus, a gene implicated in neurodevelopmental disease [32]. The latter was also identified within a MEblue module by WGCNA, in which GO enrichments to neuronal and developmental processes were shown. PGC1α is a transcription coactivator that is involved in a wide variety of biological responses including adaptive thermogenesis, mitochondrial biogenesis, glucose/fatty acid metabolism and heart development. PGC-1α is a powerful regulator of energy metabolism in both health and disease [31]; a close relationship exists among PGC-1α function, insulin sensitivity, and Type 2 diabetes. With aging, a potentially important defect has been found in the mitochondrial fatty acid oxidation pathway associated with insulin resistance [46]. *RBFOX1* gene has been recently shown to serve as a ‘hub’ in autism spectrum disorder (ASD) transcriptome networks and in neurodevelopmental and psychiatric disorder including intellectual disability (ID), attention deficit hyperactivity disorder (ADHD) and schizophrenia [32]. While there is an indication that sperm DNA methylation patterns do not persist trans-generationally [47], it has been shown that paternal age effects on sperm *FOX1* and *KCNA7* methylation can be transmitted to the next generation [48]. We speculate that age-induced hypermethylation might be related to PGC-1α dysfunction, at least in part, resulting in insulin resistance development in children of aged father, and that RBFOX1 dysfunction is implicated in the higher risk of mental disorders of children of older fathers.

Epigenetic clocks, including DNA methylation clocks, are a novel class of biomarkers of aging; they also appear to be a robust measure of chronological age in humans, and reflect the biological age of individual [49]. The best established epigenetic clocks pioneered by Hovarth [23, 50] combined blood DNA methylation values in selected sets of CpG sites in order to predict an individual’s age. Deviation of epigenetic age from chronological age (epigenetic age acceleration) has been shown to be predictive of deleterious health outcomes [51, 52]. The DNA methylation clock may not only be a measure of age, but also a regulator of age. In this study, we show a dissociation between methylome changes during aging between somatic and male germ cells. It remains to be determined whether the methylation changes found in spermatozoa during aging will be able to serve as predictors of the pattern of health and disease of men’s progeny.

## Conclusions

Using high throughput and customized MCC-seq technique, we identified thousands of novel age-related epigenomic alterations in human spermatozoa. Our results constitute important new insight on human sperm DNA methylation dynamics during the process of aging. These findings provide important new leads to determine how advanced paternal age results in DNA methylation that may have consequences on the health of their progeny.

## Methods

### Subject recruitment and study design

Both fertile and infertile subjects were recruited from the Men’s Health Clinic at the Royal Victoria Hospital, Montreal, Quebec over the period of January 2016 to December 2018. Research ethics protocol was approved by McGill University Health Centre (REB#: Human Subjects Research 15-068-MUH). All the subjects were evaluated by physicians, informed consent was obtained from all participants, with the assistance of the research nurse coordinators. Semen samples were collected by masturbation with at least 3 days abstinence. Semen parameters including semen volume, sperm count, concentration, and motility were assessed, according to the WHO guidelines [53]. Samples were aliquoted, frozen and stored at −80 °C until further analysis. Plasma hormone levels and metabolic factors were evaluated.

Inclusion criteria for fertile subjects were: (1) over the age of 18 years; (2) history of natural fecundity; (3) able to ejaculate by masturbation to provide semen samples. Exclusion criteria for fertile subjects are: (1) history of infertility (defined as inability to achieve natural conception with a reproductively health female partner after one year of unprotected sexual intercourse) at any point of his life; (2) inability to provide informed consent; (3) inadequate sperm per ejaculate (total sperm count below 5 × 10^6^ /ml).

Inclusion criteria for infertile subjects were: (1) over the age of 18 years; (2) history of idiopathic male-factor infertility (defined above), with or without anomalies in semen parameters, in the absence of identifiable causes; (3) ability to ejaculate by masturbation to provide semen samples.

For both fertile and infertile groups, the following confounding clinical conditions, known to affect spermatozoa, were used as additional exclusion criteria: recent (< 6 months) infection/inflammation of the genitourinary tract; history of hypogonadism and metabolic syndrome; varicoceles; and history of reconstructive surgery of the male reproductive tract.

In this study, 94 participants were recruited, 48 fertile controls and 46 infertile subjects. Within fertile and infertile groups, subjects were categorized in young (18–38 years) and old (46–71 years) groups.

### Extraction of genomic DNA from spermatozoa

Sperm genomic DNA (gDNA) was extracted from 5 to 10 million spermatozoa using QIAamp DNA Mini Kit (Qiagen #51306, Qiagen Mississauga, ON) according to the manufacturer’s protocols. Prior to proceeding with DNA extraction, sperm were washed using cold 0.45% NaCl for 3 times at 1500 g, 5 min at 4 °C to burst somatic cells. Sperm were then pelleted, and prior to proceeding, a visual inspection of each sample was done to ensure the absence of potentially contaminating cells. Sperm lysis was done by pipetting sperm pellets with sperm lysis buffer containing 150 mM EDTA, 10 mM Tris and 40 mM DTT, with the addition of 10% proteinase K (Sigma P4850) and 3.25% of Sarkosyl (30% N-Lauroylsarcosine sodium salt solution, Sigma 61747), and incubated at 56 °C for 2 h. Sperm DNA concentrations were quantified using a NanoDrop 2000 spectrophotometer (Thermo Fisher Scientific, Montreal, QC).

### Purity of sperm preparation assessment by pyrosequencing of imprinted gene loci

Determination of the purity of the sperm preparation, i.e., absence of contaminating somatic cells, was verified by determining DNA methylation levels of CpG dinucleotides located on the germline DMRs of maternally and paternally imprinted genes using bisulfite pyrosequencing as previously described [19] with minor modifications. Briefly, 100–500 ng of sperm DNA were subjected to bisulfite conversion treatment using the EpiTect Plus DNA Bisulfite Kit (Qiagen #59124). Pyrosequencing was done and bisulfite converted DNA as template was subject to PCR reaction using HotStartTAq Master Mix Kit (Qiagen #203443) and in a GeneAmp PCR System 2400 (Perkin Elmer, Montreal, QC). Primers were designed using Qiagen PyroMark Assay Design SW 2.0 for assessment of two paternally methylated imprinted gene loci *H19*, *DLK/GLT2 IG-DMR*, and two maternally methylated gene loci *MEST*, *KCNQ10T1.* The sequences of PCR primers and sequencing primers, and the PCR conditions are provided in Additional file 1: Table S7. Amplicons were sequenced using the PyroMark Q24 Reagents Kit (Qiagen #970802), the PyroMark Q24 Vacuum Prep Workstation and Instrument (Qiagen, Valencia, CA, USA) following the manufacturer’s protocol. All samples used in this study had methylation profiles confirming a lack of somatic cell contamination. Sperm gDNA samples (1 µg) were sent to McGill University Genome Centre and sequencing (subject for Quality Control again prior to library preparation for MethylC-capture MCC-seq).

### MethylC-capture sequencing (MCC-seq)

MCC-seq was done using a dedicated system to enrich dynamic CpG sites in human sperm as previously described [20]. WGBS and targeted bisulfite sequencing were done as previously described [29, 54]. The WGBS libraries were constructed using the KAPA High Throughput Library Preparation Kit (Roche/KAPA Biosystems) from 1 µg of the sperm DNA spiked with 0.1% (w/w) unmethylated lambda and pUC19 DNA (Promega). DNA was sonicated (Covaris) and fragments sizes of 300-400 bp were controlled on a Bioanalyzer DNA 1000 Chip (Agilent). Following fragmentation, DNA end repair of double stranded DNA breaks, 3′-end adenylation, adaptor ligation and clean-up steps were conducted according to KAPA Biosystems’ protocols. The sample was then bisulfite converted using the Epitect Fast DNA bisulfite kit (Qiagen) following manufacturer’s protocol. The resulting bisulfite DNA was quantified with OliGreen (Life Technology) and amplified with 9–12 PCR cycles using the KAPA HiFi HotStart Uracil + DNA Polymerase (Roche/KAPA Biosystems) according to suggested protocols. The final WGBS libraries were purified using Ampure Beads, validated on Bioanalyzer High Sensitivity DNA Chips (Agilent) and quantified by PicoGreen (ThermoFisher). For WGBS libraries the SeqCap Epi Enrichment System protocol (Roche NimbleGen) was used to capture the regions of interest. Equal amounts of multiplexed libraries (84 ng of each; 12 samples per capture) were combined to obtain 1 µg of total input library and was hybridized to the capture panel at 47 °C for 72 h. Washing, recovery, PCR amplification of the captured libraries as well as final purification were conducted as recommended by the manufacturer. Bioanalyzer High Sensitivity DNA Chips (Agilent) were used to determine quality, concentration and size distribution of the final captured libraries. The capture libraries were sequenced with a 200-cycle S2 kit (100-bp paired end) on the NovaSeq 6000 following the NovaSeq XP workflow, at a depth of 0.5 lanes per library.

### Sequencing data processing

Targeted sperm panel MCC-Seq HiSeq reads were aligned using the Epigenome Pipeline available from the DRAGEN Bio-IT platform (Edico Genomics/Illumina). Specifically, the MCC-Seq paired-end raw reads were first demultiplexed into FASTQ files using Illumina's bcl2Fastq2-2.19.1 software. Reads were then trimmed for quality (phred33 ≥ 20) and Illumina adapters using trimgalore v.0.4.2, a wrapper tool around Cutadapt [55] and FastQC. Then the trimmed reads were aligned, to the bisulfite-converted GRCh37 reference genome using DRAGEN EP v2.6.3 or later in paired end mode using the directional/Lister methylation protocol presets; alignments were calculated for both Watson and Crick strands and the highest quality unique alignment was retained. A genome-wide cytosine methylation report was generated by DRAGEN to record counts of methylated and unmethylated cytosines at each cytosine position in the genome. Methylation counts are provided for the CpG, CHG and CHH cytosine contexts. DNA methylation level of each CpG was calculated by the number of methylated reads over the total number of sequenced reads. CpGs that were found to be overlapping with SNPs (dbSNP 137), the DAC Blacklisted Regions or Duke Excluded Regions (generated by the ENCODE project) were removed. CpG sites with less than 20× read coverage were also discarded.

### Age prediction analysis

Sperm age predictor for discovery dataset was built using the R package glmnet [56]. Starting with the top 5000 significant DMCs, CpGs which were covered by all samples in the discovery cohort were selected to build the predictor. The DNA methylation level was ranged from 0 to 1. Similar to Horvath’s strategy, the alpha parameter of glmnet was chosen to 0.5 (elastic net regression) and the lambda value was chosen using leave-one-out cross-validation (LOOCV) on the training data [23].

### Genomic enrichment analyses

We utilized Genomic Regions Enrichment of Annotations Tool (GREAT) [33] to explore general characteristics of CpGs associated with sperm age in a random set of 88,000 CpGs targeted for custom capture and sequencing [20] as background. To characterize gene networks potentially linked to age-associated CpGs, we fetched ENSEMBL gene annotations for significantly GREAT enriched “foreground” regions (as compared to all targeted CpGs). The analysis of enriched gene ontologies among GREAT significant “foreground” gene regions was then pursued by selecting genes with significantly increased density (± 1 Mb) of multiple testing (qv < 0.01) adjusted hypo- or hypermethylated CpGs as compared to random (targeted) CpG background. To further identify the hypo- and hyper-DMC relative density, we calculated the ratio of DMCs over the total number of CpGs within 1 Mb sliding windows over the genome.

We also performed GO analyses using Metascape [57] to enrich genes in age dependent hypo- and hypermethylated regions. We investigated the distribution of chromatin states (ChromHMM from ENCODE data) of sperm age-related hypo- and hypermethylated CpGs in human hESC (H1) [34].

Evolutionarily constraint among hypo- and hypermethylated age-CpGs was done using genomic element rate profiling (GERP) analysis, and evolutionary constrained hypo- and hypermethylated DMRs were localized to disease (OMIM) relevant gene loci [58].

### Weighted gene-co-expression network analysis (WGCNA)

Weighted gene co-expression network analysis (WGCNA), aimed at detecting highly correlated module of CpGs, was done using 20,000 variable CpGs. The input CpGs were chosen based on uniform (no missing values) coverage (i.e. ≥20X) and population variance (i.e. top 20,000 most variable CpGs based on their variance, and roughly top 5% of variance) among our samples. The WGCNA was ran using the WGCNA package in R [38] with the default parameters. A soft-thresholding power of 8, which is the lowest power for which the scale-free topology fit index curve flattens out upon reaching a high value of signed R-square of 0.9, was chosen. A maximum block size of 20,000 and unsigned network were used (to construct a topological overlap matrix) and minimum number of CpGs per module = 30 and cut height = 0.4 were used to detect methylation modules. The module eigengene (ME) value was calculated for each module and further the Pearson correlation between MEs and traits was computed to quantify module-trait associations.

The CpGs from significant module trait correlations (*p* < 0.005, absolute correction |Corr|> 0.3) were used as input to GREAT protein coding gene enrichment using top 20 K variable CpGs as above. For the top 2 most complex modules involving 100 genes, we further performed GO enrichment analyses.

### Statistical analyses

Statistical analyses for the characterization of subjects were done using GraphPad Prism 6 Software (San Diego, CA). Comparisons of semen parameters, biochemical and metabolic factors between fertile and infertile subjects were done using Student’s *t*-test. Pearson correlations were used for age and other semen parameters and metabolic factors. *p* < 0.05 was considered statistically significant.

Linear regression models (LMs) were built to investigate the association between the DNA methylation level (methylation proportion defined above) and age. The age LM model was adjusted by correcting infertility status, sperm concentration, smoking status, and other clinic indices such as total testosterone, bioavailable testosterone concentrations, plasma FSH level, high density lipoprotein (HDL), and other metabolic parameters including BMI, plasma triglycerides and the risk ratio of total cholesterol to HDL. We used the R function lm() to fit the models, and calculated p-values for variables of interest. QQ-plot and Manhattan plot were reported for each LM. We further evaluated the obtained p-values by generating false discovery proportion q-values using the R package q-values [59]. We reported qv < 0.01 as the genome-wide significant DMCs. The detailed number of CpGs used in each model is listed in Additional file 1: Table S2.

## Supplementary information

**Additional file 1: Table S1**. Pearson correlation analysis of the age of subjects and clinical indices in the fertile and infertile subjects. **Table S2**. Association results for age models with different corrections. **Table S3**. Chromatin states distribution (ChromHMM from ENCODE data) of sperm age hypo –and hypermethylated regions in human hESC (H1). **Table S7**. Bisulfite pyrosequencing primers and PCR conditions for human sperm germline differentially methylated regions (DMRs)

**Additional file 2: Table S4**. Age related hypermethylated DMRs and closest genes with OMIM genes annotated.

**Additional file 3: Table S5**. Enriched gene lists by GREAT in hyper- or Hypomethylated regions.

**Additional file 4: Table S6**. WGCNA module enriched genes and CpGs.

**Additional file 5: Figure S1**. DNA methylation profiles of imprinted gene loci approved sperm DNA sample purity. Two paternal methylated gene loci **a** H19 and **b** DLK1/GTL2 IG-DMR, two maternal methylated gene loci **c** MEST and **d** KCNQ10T1. Blue area in each genes indicated the CpG sites examined in pyrosequencing analysis. Sperm DNA methylation of each individual by MCC-seq further validated the pyrosequencing results. **Figure S2**. QQ-plots for associations tests. Unadjusted association tests for** a** age and** b** fertility show excess of age associations (black dots) above null expectation (red line), whereas for fertility no associations survive multiple testing correction. **Figure S3**. Genome-wide density of hyper/hypomethylated regions upon sperm aging. Density of hypomethylation is relatively even across the genome. Overall, sex chromosomes are relatively depleted of age associated CpGs. **Figure S4**. Genomic element rate profiling (GERP) analysis shows evolutional constrain is similar among hyper-and hypomethylated CpGs. X-axis shows data deciles and Y-axis GERP++ scores for hypermethylated (blue) and hypomethylated CpGs as function of age. Approximately 15% of CpGs lie in areas showing similar constraint as known functional elements (GERP++ > 1.7) (PMID: 21152010). **Figure S5**. Heatmap of module-trait relationships revealed by WGCNA analysis. Forty-three module eigengenes (MEs) listed in different colors on the left, were correlated to age, infertility, smoking and BMI traits shown on the bottom. The average module-trait correlation values and p-values were indicated individually, correlation value scale was shown on the right. Note that two highly significant modules each linked to age, and one moderate but highly significant module was associated with infertility trait. **Figure S6**. GO enrichments for genes loci near age-linked CpG modules identified by WGCNA. Both **a** MEblue (corr = 0.62, P = 2.00E-11) and **b** ME brown (corr = −0.62, P = 3.00E-11) modules show GO enrichment to neuronal as well as developmental processes with significant age association.

## Data Availability

The raw reads data generated by MCC-seq during the current study have been submitted to the European Genome-phenome Archive under the accession number EGAS00001004168.

## Abbreviations

ADHD: Attention deficit hyperactivity disorder
ASD: Autism spectrum disorder
BMI: Body mass index
CNS: Central nervous system
DMCs: Differentially methylated CpG sites
DMRs: Differentially methylated regions
FSH: Follicle stimulating hormone
GERP: Genomic element rate profiling
GO: Gene Ontology
GREAT: Genomic regions enrichment of annotations tool
HDL: High density lipoprotein
ID: Intellectual disability
IVF: In vitro fertilization
LDL: Low density lipoprotein
LH: Luteinizing hormone
LOOCV: Leave-one-out cross-validation
MCC-seq: MethylC-capture sequencing
ME: Module eigengene
PGC-1α: Peroxisome proliferator-activated receptor (PPAR)-gamma coactivator 1-alpha
RRBS: Reduced representation bisulfite sequencing
RBFOX1: RNA binding fox-1 homolog 1
TSH: Thyroid stimulating hormone
TSS: Transcription start site
WGBS: Whole genome bisulfite sequencing
WGCNA: Weighted gene co-expression network analysis
