# Supplementary material for: High-resolution analyses of human sperm dynamic methylome reveal thousands of novel age-related epigenetic alterations
Source: Clin Epigenetics. 2020 Dec 14;12:192. doi: 10.1186/s13148-020-00988-1 (PMC7735420; doi:10.1186/s13148-020-00988-1)
Supplement: Supplementary file 1 — Additional file 1: Table S1. Pearson correlation analysis of the age of subjects and clinical indices in the fertile and infertile subjects. Table S2. Association results for age models with different corrections. Table S3. Chromatin states distribution (ChromHMM from ENCODE data) of sperm age hypo –and hypermethylated regions in human hESC (H1). Table S7. Bisulfite pyrosequencing primers and PCR conditions for human sperm germline differentially methylated regions (DMRs) [file 13148_2020_988_MOESM1_ESM.docx]

**Additional file 1:**

**Table S1. Pearson correlation analysis of the age of subjects and clinical indices in the fertile and infertile subjects**

|  |  | **Fertile** |  |  | **Infertile** |  |
| --- | --- | --- | --- | --- | --- | --- |
| **Parameters** | **r** | **P** | **N** | **r** | **P** | **N** |
| Age vs. sperm count | 0.24 | 0.10 | 48 | 0.13 | 0.39 | 45 |
| Age vs. motility | -0.26 | 0.08 | 48 | -0.29 | 0.048* | 46 |
| Age vs. FSH | 0.71 | < 0.0001**** | 48 | 0.24 | 0.11 | 45 |
| Age vs. LH | 0.18 | 0.22 | 48 | 0.04 | 0.79 | 44 |
| Age vs. TSH | 0.17 | 0.28 | 44 | 0.16 | 0.32 | 43 |
| Age vs. E2RP | 0.14 | 0.43 | 35 | 0.01 | 0.94 | 31 |
| Age vs. Testosterone (T) | -0.39 | 0.006* | 48 | -0.27 | 0.07 | 45 |
| Age vs. Bio T | -0.63 | <0.0001**** | 46 | -0.47 | 0.0012** | 44 |
| Age vs. BMI | 0.42 | 0.003** | 48 | 0.09 | 0.56 | 46 |
| Age vs. Total Cholesterol | 0.21 | 0.17 | 46 | 0.12 | 0.44 | 45 |
| Age vs. Triglycerides | 0.34 | 0.02* | 46 | 0.20 | 0.20 | 44 |
| Age vs. HDL | -0.38 | 0.009** | 45 | 0.06 | 0.72 | 44 |
| Age vs. LDL | 0.14 | 0.37 | 41 | 0.14 | 0.39 | 42 |
| Age vs. Cholesterol  /HDL risk ratio | 0.37 | 0.013* | 45 | -0.006 | 0.97 | 44 |

FSH: follicle stimulating hormone; LH: luteinizing hormone; TSH: thyroid stimulating hormone; E2RP: estradiol levels; BMI: body mass index; HDL: high density lipoprotein; LDL: low density lipoprotein.

**Table S2. Association results for age models with different corrections**

|  | **#TestedCpGs** | **#DMC_qv<0.01** |
| --- | --- | --- |
| LM.Age | 2654805 | 155416 |
| LM.Fertility | 2654805 | 0 |
| LM.Age+Fertility+all covariates | 2599141 | 21971 |

**Table S3. Chromatin states distribution (ChromHMM from ENCODE data) of sperm age hypo –and hypermethylated regions in human hESC (H1)**

| **ESC/H1 chromHMM state** | **Hypo** | **Hyper** |
| --- | --- | --- |
| 1_Active_Promoter | 0.2% | 0.0% |
| 2_Weak_Promoter | 4.6% | 0.7% |
| 3_Poised_Promoter | 14.6% | 0.7% |
| 4_Strong_Enhancer | 2.7% | 0.4% |
| 5_Strong_Enhancer | 1.5% | 0.2% |
| 6_Weak_Enhancer | 8.7% | 3.2% |
| 7_Weak_Enhancer | 7.8% | 7.7% |
| 8_Insulator | 3.6% | 3.1% |
| 9_Txn_Transition | 4.1% | 0.0% |
| 10_Txn_Elongation | 1.7% | 0.0% |
| 11_Weak_Txn | 15.5% | 11.0% |
| 12_Repressed | 12.9% | 1.3% |
| 13_Heterochrom/lo | 19.9% | 71.4% |
| 14_Repetitive/CNV | 2.2% | 0.4% |

**Table S7. Bisulfite pyrosequencing primers and PCR conditions for human sperm germline differentially methylated regions (DMRs)**

| **Germline DMR (No. of CpGs examined)** | **Methylated Allele** | **Bisulfite converted Sequence (5'-3')**^a^ | **Location**^b^ | **PCR amplicon (bp)** | **PCR conditions (annealing temperature)** |
| --- | --- | --- | --- | --- | --- |
| ***H19***  ***(7)*** |  | **F:**GGGATGTTTTTGTAGGTAAATTATAGGTAG | chr11:2,022,355-2,022,636 |  | 55.6°C |
|  | Paternal | **R*:**ACTTCCAAAATAAAAAATCCCTATATTCTA |  | 282 |  |
|  |  | **S_1_:**TTGTAGGTAAATTATAGGTAGT |  |  |  |
|  |  | **S_2_**:TTGTGTTTTTATATTTTTAATTGAT |  |  |  |
| ***DLK1/GTL2* IG-DMR *(6)*** |  | **F:**AGTAGTTGGGATTATAGGTATTATGT | chr14:101,276,661-101,276,851 |  | 58°C |
|  | Paternal | **R*:**ATCTTTTAAACTCTACCCTATCTTACTCT |  | 217 |  |
|  |  | **S:** GATAATTTTTTGTATTTTTAGTAGG |  |  |  |
| ***MEST (6)*** |  | **F*:**TTATTAGTTTGGTGGTGGGTTTAATAG | chr7:130,131,314-130,131,616 |  | 55.6°C |
|  | Maternal | **R:** CCCTAAATACCCCAACTCTT |  | 303 |  |
|  |  | **S:**CTAAAAAACAACAAAACTCTAAAAT |  |  |  |
| ***KCNQ1OT1 (15)*** |  | **F:** AGGGGAGTTTTGGTAGGATTTTGT | chr11:2,721,153-2,721,466 |  | 65°C |
|  | Maternal | **R*:**CCTCACACCCAACCAATACCTCATA |  | 314 |  |
|  |  | **S_1_:** GGAGTTTTTTGGAGG |  |  |  |
|  |  | **S_2_**: GTGGAGATAGTTTTTTATTTGT |  |  |  |

^a^ Primers indicated by a star are biotinylated at the 5' end. (Forward primer has no C, reverse primer has no G after bisulfite conversion.)

^b^ Chromosomal location based on Ensembl release 76 - August 2014. (Aarabi M et al., 2015)
