# Supplementary material for: High-resolution analyses of human sperm dynamic methylome reveal thousands of novel age-related epigenetic alterations
Source: Clin Epigenetics. 2020 Dec 14;12:192. doi: 10.1186/s13148-020-00988-1 (PMC7735420; doi:10.1186/s13148-020-00988-1)
Supplement: Supplementary file 5 — Additional file 5: Figure S1. DNA methylation profiles of imprinted gene loci approved sperm DNA sample purity. Two paternal methylated gene loci a H19 and b DLK1/GTL2 IG-DMR, two maternal methylated gene loci c MEST and d KCNQ10T1. Blue area in each genes indicated the CpG sites examined in pyrosequencing analysis. Sperm DNA methylation of each individual by MCC-seq further validated the pyrosequencing results. Figure S2. QQ-plots for associations tests. Unadjusted association tests for a age and b fertility show excess of age associations (black dots) above null expectation (red line), whereas for fertility no associations survive multiple testing correction. Figure S3. Genome-wide density of hyper/hypomethylated regions upon sperm aging. Density of hypomethylation is relatively even across the genome. Overall, sex chromosomes are relatively depleted of age associated CpGs. Figure S4. Genomic element rate profiling (GERP) analysis shows evolutional constrain is similar among hyper-and hypomethylated CpGs. X-axis shows data deciles and Y-axis GERP++ scores for hypermethylated (blue) and hypomethylated CpGs as function of age. Approximately 15% of CpGs lie in areas showing similar constraint as known functional elements (GERP++ > 1.7) (PMID: 21152010). Figure S5. Heatmap of module-trait relationships revealed by WGCNA analysis. Forty-three module eigengenes (MEs) listed in different colors on the left, were correlated to age, infertility, smoking and BMI traits shown on the bottom. The average module-trait correlation values and p-values were indicated individually, correlation value scale was shown on the right. Note that two highly significant modules each linked to age, and one moderate but highly significant module was associated with infertility trait. Figure S6. GO enrichments for genes loci near age-linked CpG modules identified by WGCNA. Both a MEblue (corr = 0.62, P = 2.00E-11) and b ME brown (corr = −0.62, P = 3.00E-11) modules show GO enrichment to neuron [file 13148_2020_988_MOESM5_ESM.pptx]

## Slide 1
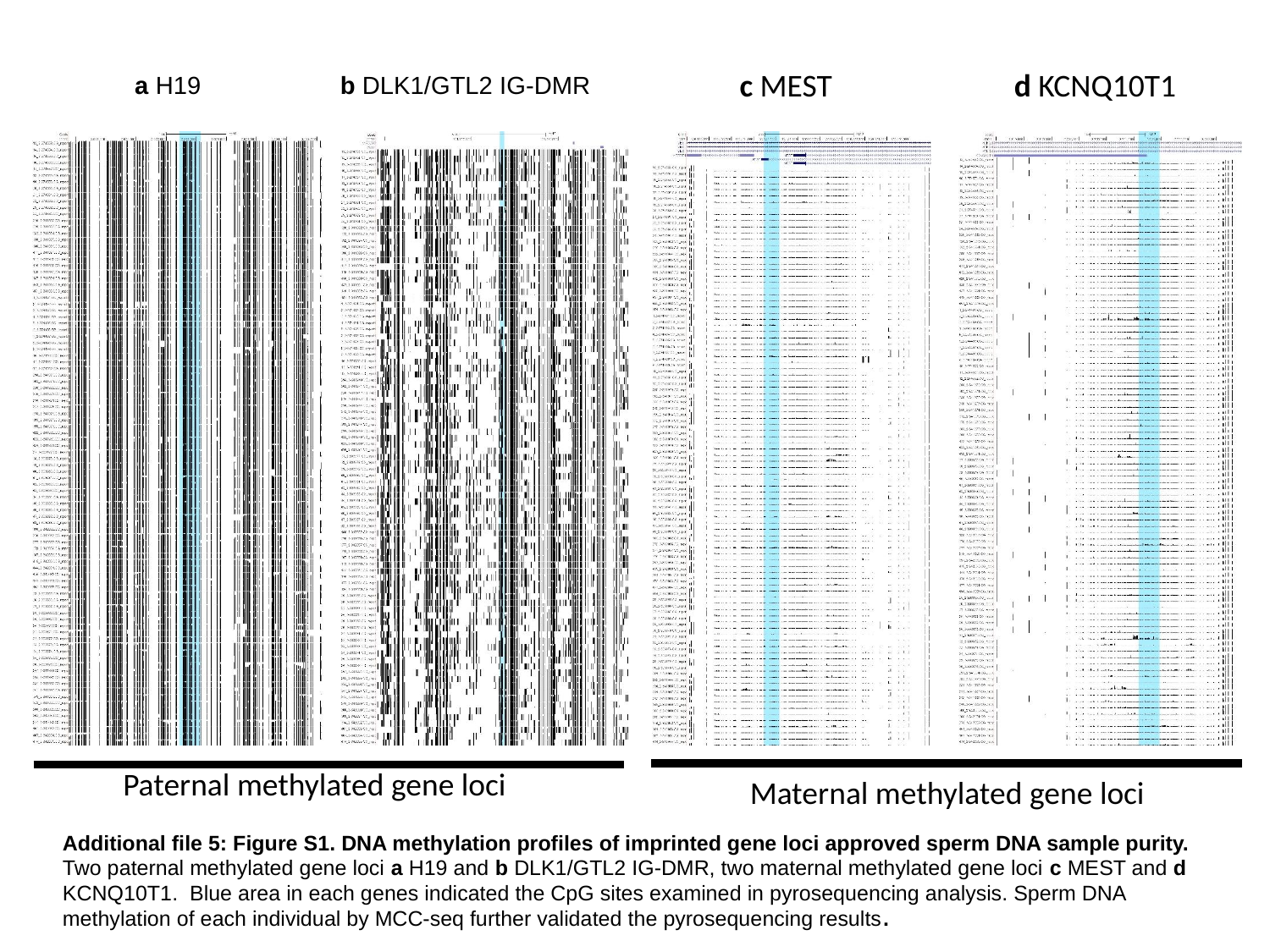

c MEST
d KCNQ10T1
a H19
b DLK1/GTL2 IG-DMR
Paternal methylated gene loci
Maternal methylated gene loci
Additional file 5: Figure S1. DNA methylation profiles of imprinted gene loci approved sperm DNA sample purity. Two paternal methylated gene loci a H19 and b DLK1/GTL2 IG-DMR, two maternal methylated gene loci c MEST and d KCNQ10T1. Blue area in each genes indicated the CpG sites examined in pyrosequencing analysis. Sperm DNA methylation of each individual by MCC-seq further validated the pyrosequencing results.

## Slide 2
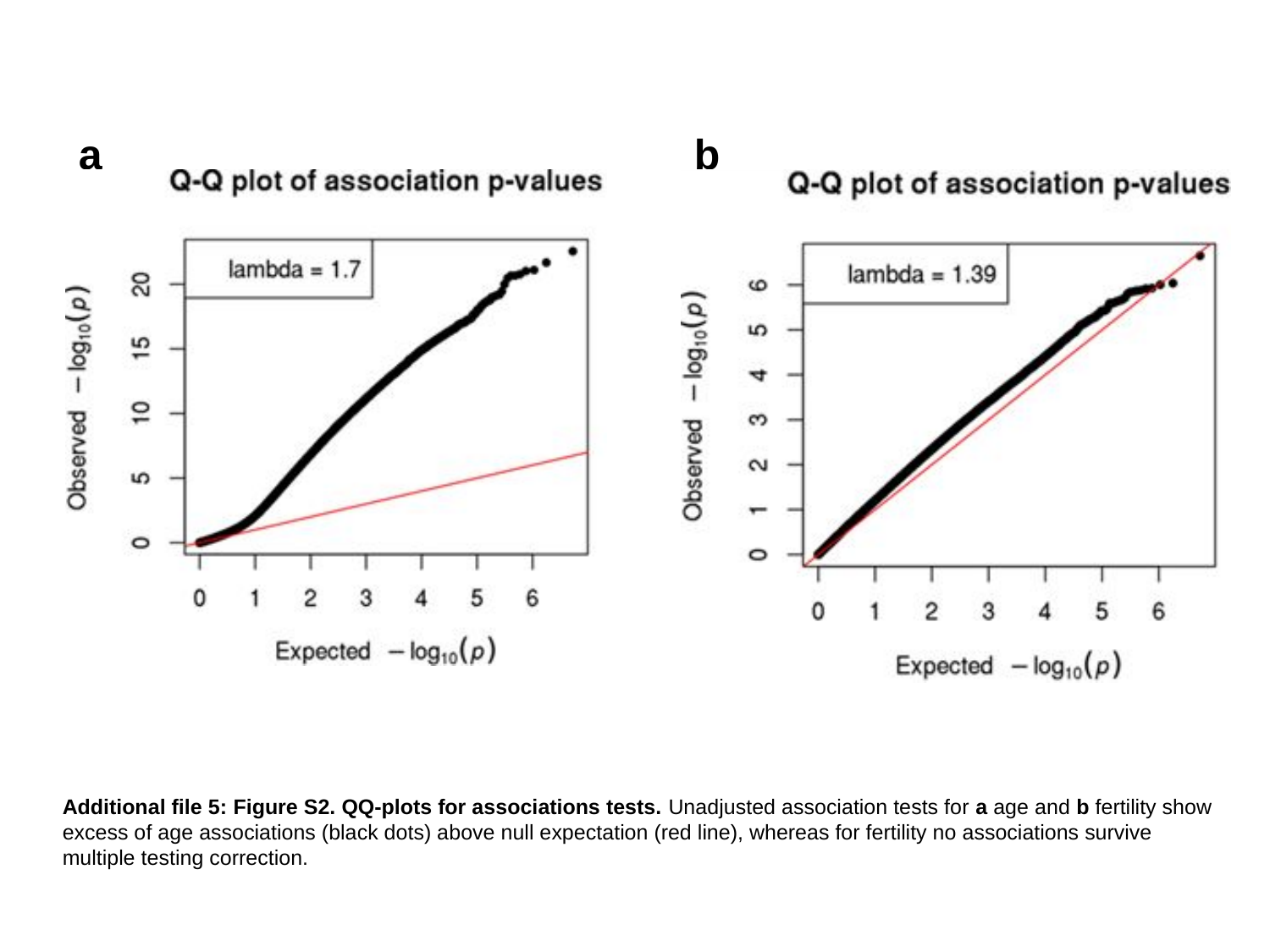

a
b
Additional file 5: Figure S2. QQ-plots for associations tests. Unadjusted association tests for a age and b fertility show excess of age associations (black dots) above null expectation (red line), whereas for fertility no associations survive multiple testing correction.

## Slide 3
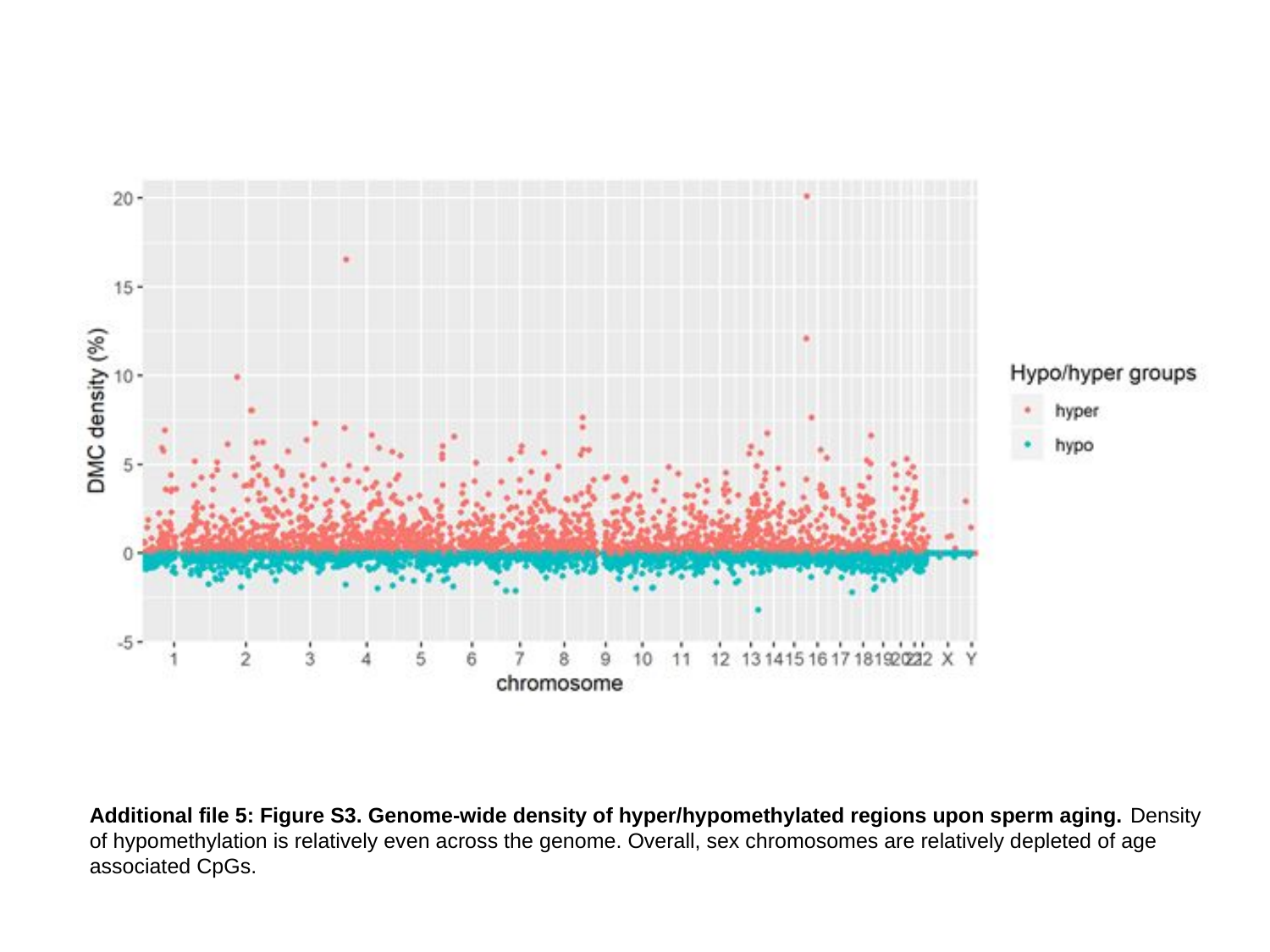

Additional file 5: Figure S3. Genome-wide density of hyper/hypomethylated regions upon sperm aging. Density of hypomethylation is relatively even across the genome. Overall, sex chromosomes are relatively depleted of age associated CpGs.

## Slide 4
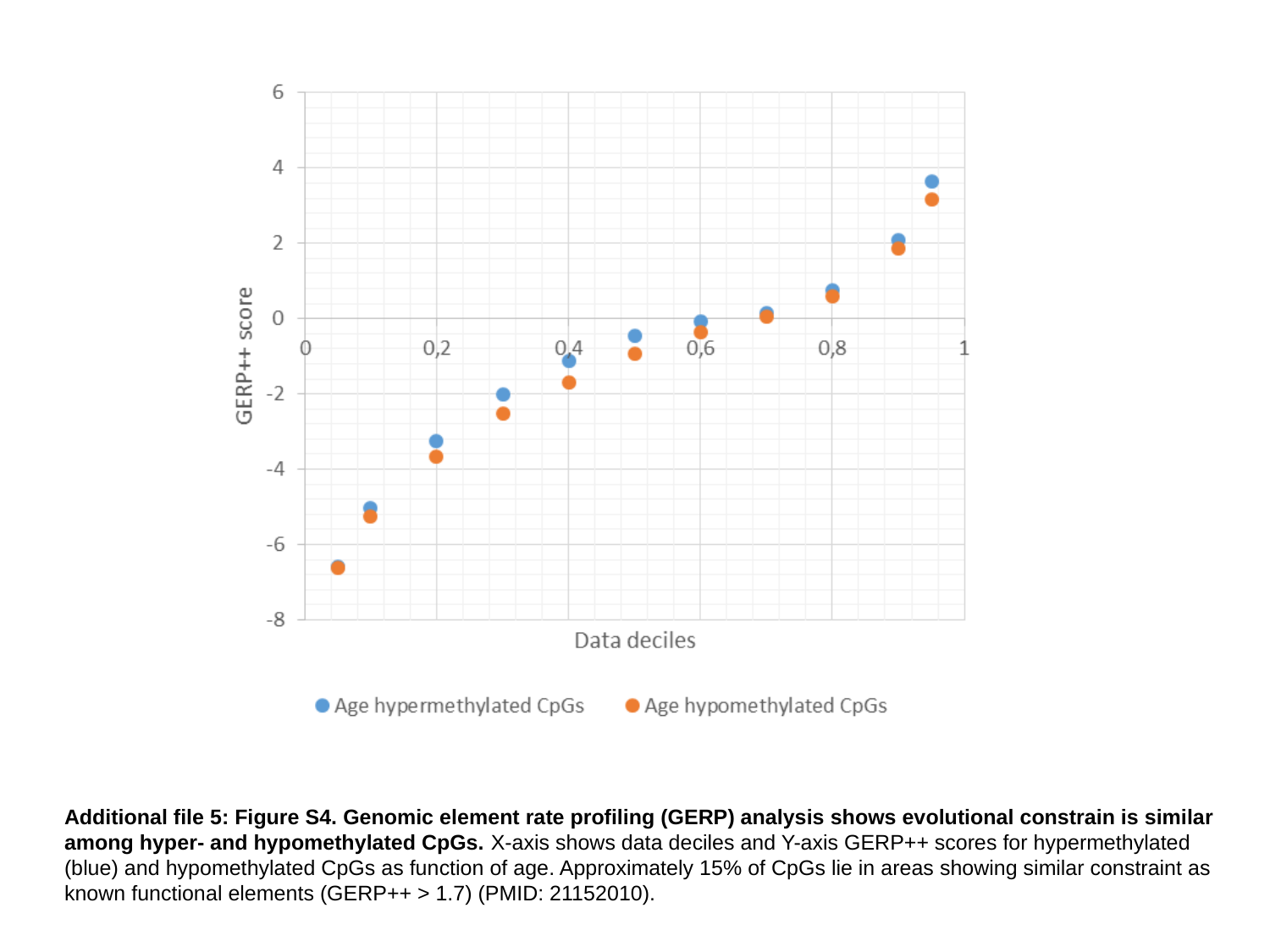

Additional file 5: Figure S4. Genomic element rate profiling (GERP) analysis shows evolutional constrain is similar among hyper- and hypomethylated CpGs. X-axis shows data deciles and Y-axis GERP++ scores for hypermethylated (blue) and hypomethylated CpGs as function of age. Approximately 15% of CpGs lie in areas showing similar constraint as known functional elements (GERP++ > 1.7) (PMID: 21152010).

## Slide 5
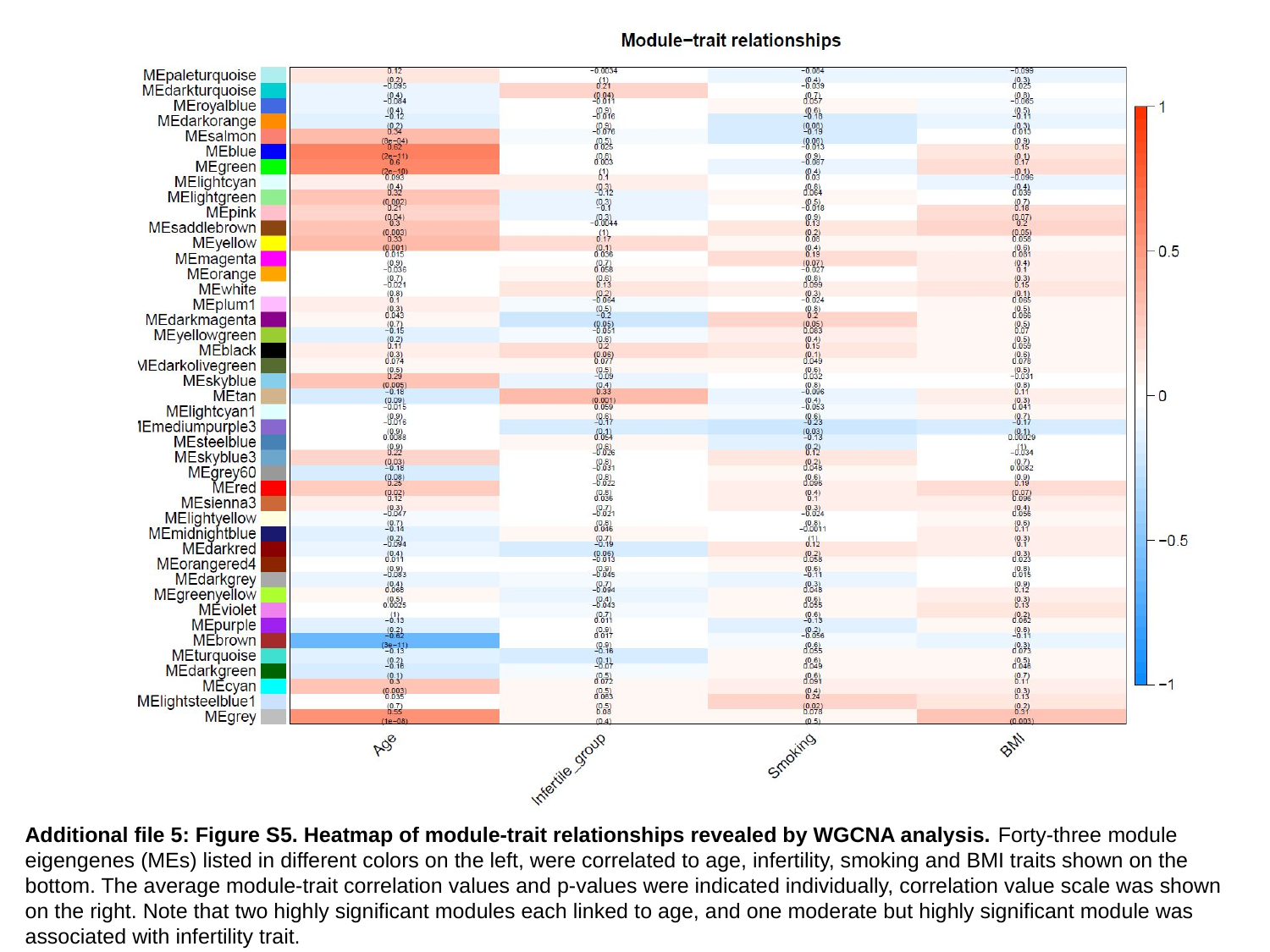

Additional file 5: Figure S5. Heatmap of module-trait relationships revealed by WGCNA analysis. Forty-three module eigengenes (MEs) listed in different colors on the left, were correlated to age, infertility, smoking and BMI traits shown on the bottom. The average module-trait correlation values and p-values were indicated individually, correlation value scale was shown on the right. Note that two highly significant modules each linked to age, and one moderate but highly significant module was associated with infertility trait.

## Slide 6
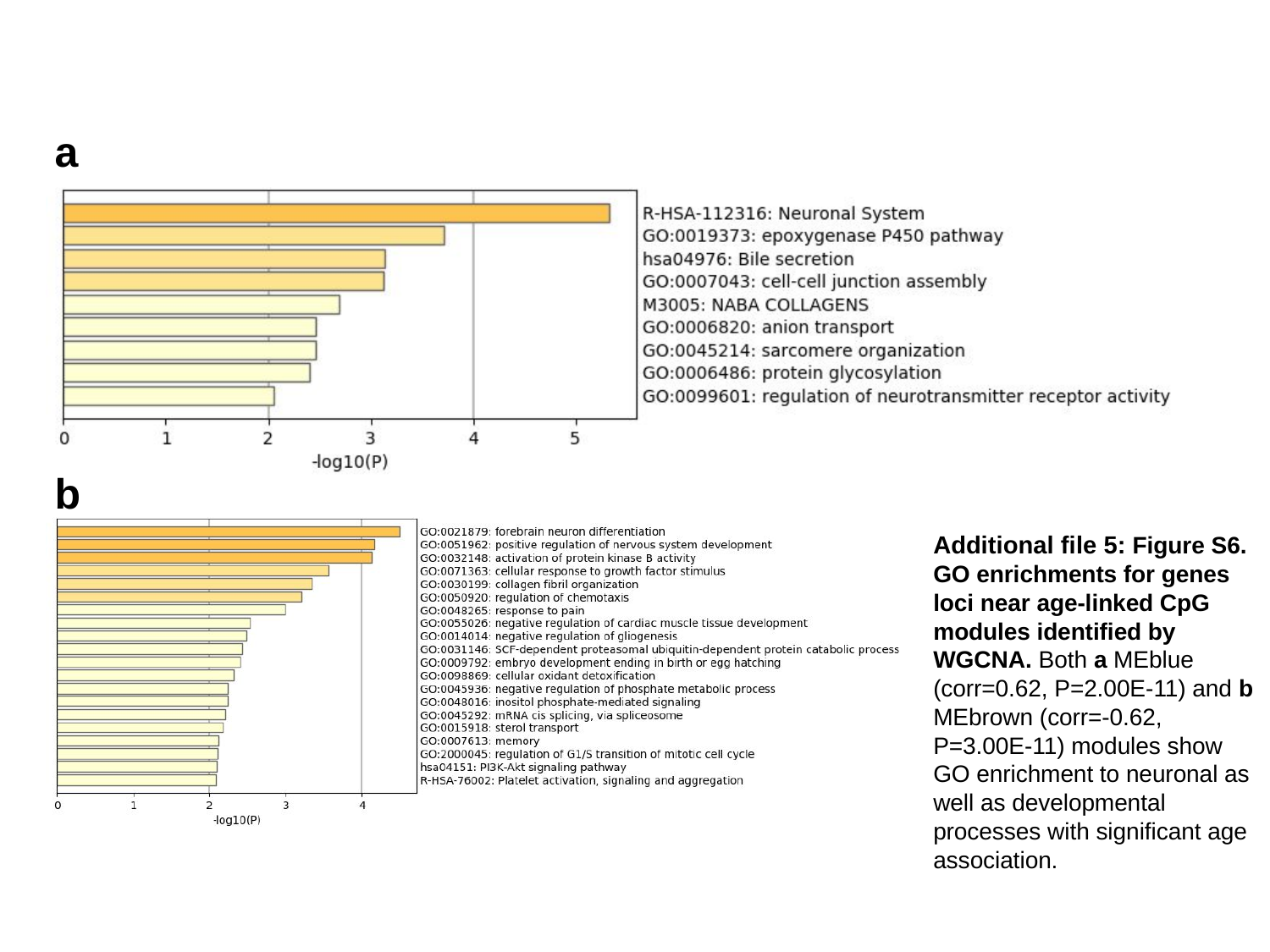

a
b
Additional file 5: Figure S6.
GO enrichments for genes loci near age-linked CpG modules identified by WGCNA. Both a MEblue (corr=0.62, P=2.00E-11) and b MEbrown (corr=-0.62, P=3.00E-11) modules show GO enrichment to neuronal as well as developmental processes with significant age association.
